# Association of B-Type Natriuretic Peptide Level with Clinical Outcome in Out-of-Hospital Cardiac Arrest in Emergency Department Patients

**DOI:** 10.3390/diagnostics13152522

**Published:** 2023-07-28

**Authors:** Heejin Hong, Jihyun Kim, Hana Min, Yong Won Kim, Tae-Youn Kim

**Affiliations:** 1 Department of Emergency Medicine, Dongguk University Ilsan Hospital, Dongguk University College of Medicine, 27, Dongguk-ro, Ilsandong-gu, Goyang 10326, Republic of Korea; 2 Department of Emergency Medicine, National Health Insurance Service Ilsan Hospital, 100, Ilsan-ro, Ilsandong-gu, Goyang 10444, Republic of Korea

**Keywords:** biomarker, cardiopulmonary resuscitation, b-type natriuretic peptide, outcome assessment

## Abstract

Objectives: B-type natriuretic peptide (BNP) is used for outcome assessment of various diseases. We designed this study to investigate whether BNP, which has been proven useful in the risk stratification of sudden cardiac arrest (SCA) of cardiac etiology, can also prove to be a valuable prognostic tool for SCA also included with non-cardiac etiology. In this study, we aim to investigate the relationship between measured BNP levels and clinical outcomes in SCA, regardless of the cause of SCA. Methods: This retrospective multicenter observational study was performed in two tertiary university hospitals and one general hospital between January 2015 and December 2020. The total number of SCA patients was 1625. The patients with out-of-hospital cardiac arrest over 19 years old and acquired laboratory data, including BNP at emergency department (ED) arrival, were included. BNP was measured during advanced Cardiovascular Life Support (ACLS). The exclusion criteria were age under 18 years, traumatic arrest, and without BNP. Results: The median BNP was 171.8 (range; 5–5000) pg/mL in the return of Spontaneous Circulation (ROSC), higher than No-ROSC (*p* = 0.007). The median BNP concentration was 99.7 (range; 5–3040.68) pg/mL in the survival to discharge, which was significantly lower than the death group (*p* = 0.012). The odds ratio of survival to discharge decreased proportionally to the BNP level. The odds ratio of neurologic outcome was not correlated with the BNP level. Conclusion: In patients with SCA of all origins, low BNP concentration measured during ACLS correlated with an increased ratio of survival to discharge. However, BNP measured during ACLS was not found to be an independent factor.

## 1. Introduction

Sudden cardiac arrest (SCA) occurs in more than 360,000 people per year in the United States [1]. A large number of out-of-hospital cardiac arrest (OHCA) cases occur annually, and the worldwide survival rate is less than 7–12% [2]. In Korea, the recently reported incidence rate of OHCA was 44.4 per 100,000 persons, and similar results have been reported by other countries [3]. SCAs are further subdivided etiologically into cardiac and noncardiac causes. Noncardiac causes include non-traumatic hemorrhage, sepsis, anesthetic- and medication-related complications, massive pulmonary embolism, and vascular collapse (e.g., anaphylaxis, amniotic fluid embolism) [4,5]. SCAs of cardiac etiology have better survival and neurologic outcomes at discharge than SCAs of noncardiac causes [5,6]. The prognosis of SCAs can help physicians during triage, treatment, and overall related management [7]. Biomarkers represent an area of growing interest in this field as they may provide clinicians with early information on the severity of organ dysfunction and enable them to make decisions on clinical strategies and evaluate potential outcomes [8,9]. Similar to worldwide findings, noncardiac causes of SCA are more common in Korea [10]. Several scoring systems have been devised to predict the outcome of SCAs; however, a multidisciplinary approach incorporating neurological examination, electroencephalography, biomarkers, and brain imaging is recommended because there is no single reliable prognostic factor [11,12]. Previous studies have devised NSE and S100B to predict brain injury, which are used to predict neurological outcomes after targeted temperature management [13]. Other biomarkers are those related to systemic inflammatory response, such as procalcitonin and C-reactive protein [14,15]. Elevated PCT within the first 24 h is an independent marker associated with mortality [16]. Troponin and creatine kinase are commonly used biomarkers after cardiovascular ischemic events, including SCA [17]. In addition, the changes in cardiomyocytes that occur during SCA result in the release of natriuretic peptides, particularly brain natriuretic peptide (BNP). BNP is commonly used in the diagnosis, treatment monitoring, and follow-up of several clinical conditions, such as heart failure, weaning from mechanical ventilation, and acute respiratory distress [18]. BNP is used for outcome assessment of various diseases other than heart disease [19,20]. This study was designed to investigate whether BNP could be a useful prognostic tool in patients with out-of-hospital cardiac arrest of any cause.

To our knowledge, the prognostic value of BNP in cardiac arrest, regardless of etiology, is yet to be explored. Thus, in this study, we aim to investigate the relationship between measured BNP levels and clinical outcomes in SCA, regardless of the cause of SCA.

## 2. Methods

### 2.1. Study Design and Setting

This multicenter, retrospective, observational study involved two tertiary university hospitals and one general hospital. The study duration was between January 2015 and December 2020. This study was approved by the Institutional Review Board (IRB) of Wonju Severance Christian Hospital (IRB no. CR322004), the IRB of Dongguk University (IRB No. DUIH 2022-02-027), and the IRB of National Health Insurance Service Ilsan Hospital (IRB No. NHIMC 2022-03-030-001). Informed consent was waived because of the retrospective nature of the study, and the analysis used anonymous clinical data.

### 2.2. Participants

All OHCA patients aged 19 years and older who visited the emergency department from January 2015 to December 2020 were included. The total number of patients visiting the ED was 1625. All patients were treated according to current advanced cardiac life support (ACLS) guidelines and were evaluated to identify reversible causes of cardiac arrest within at least 2–3 cycles of CPR. Exclusion criteria were age less than 18 years, traumatic arrest, and absence of BNP.

### 2.3. Study Variables

Clinical and laboratory parameters were retrospectively obtained from medical records, including age, sex, witness of cardiac arrest, bystander CPR, initial shockable rhythm, out-of-hospital CPR time, in-hospital CPR time, total CPR time, total adrenaline (epinephrine) dose administered, BNP levels, return of spontaneous circulation (ROSC), survival to discharge, and neurological outcome evaluated at hospital discharge. Initial shockable rhythm, out-of-hospital CPR time, and bystander CPR were documented in the medical record from the EMS transporting the patient. BNP levels were measured to determine the cause of reversible cardiac arrest according to cardiopulmonary resuscitation (CPR) guidelines within at least 2–3 cycles during CPR. The etiology of cardiac arrest was retrospectively collected based on the circumstances of cardiac arrest and death certificates for those who did not achieve ROSC or who did not achieve survival to discharge. The etiology of cardiac arrest of survival to discharge patients was collected by two doctors through medical records.

### 2.4. Measurement of Blood BNP Concentration

All blood BNP concentrations were measured during ACLS within at least 2–3 cycles during CPR. Quantitative values were calculated using a fluorescence immunoassay BNP kit (Triage^®^, Biosite, San Diego, CA, USA) after collecting 3–5 mL of blood in a test tube containing whole blood or a minimal amount of EDTA. The lower limit was 5 pg/mL, and the upper limit was 5000 pg/mL. Patients whose NT-pro BNP was measured using different methods in different periods and institutions were excluded. 

### 2.5. Study Endpoints

The primary outcome was ROSC. ROSC was defined as sustained ROSC that lasted more than 20 min. The secondary outcome was survival to discharge (including home discharge and nursing home discharge) for patients with ROSC; however, we excluded patients transferred to other institutions and patients with unknown follow-up. Neurological outcome was considered as a tertiary outcome in patients who survived to discharge and was graded using Cerebral Performance Categories (CPCs). CPC was measured by two or more physicians at the time of final discharge. A good neurologic outcome received a CPC score of 1–2, whereas a poor neurologic outcome received a CPC score of 3–5. 

### 2.6. Statistical Analysis

Continuous data are shown as means with standard deviation or median with interquartile range, followed by a normality test (such as the Shapiro–Wilk test). Categorical variables are shown as counts and percentages. Continuous data were analyzed using Student’s *t* or Mann–Whitney U tests, as appropriate. Categorical data were analyzed using Chi-square or Fisher’s exact tests as appropriate. Univariate and multivariate logistic regression analyses were performed to evaluate factors contributing to clinical outcomes, including ROSC, survival to hospital discharge, and favorable neurological outcome, with odds ratios (ORs) and 95% confidence intervals (CIs) presented. To compare the predictive ability of BNP, receiver operating characteristic curves were created using optimal cutoff values determined using the Youden index. Variables with a *p*-value < 0.2 in the univariate logistic regression analysis were included in the multivariate logistic regression analysis. In addition to analyzing BNP as a continuous variable, we also grouped BNP levels into quartiles: patients were divided into four groups based on quartiles of BNP concentration. We used one-way ANOVA for continuous variables and the Kruskal–Wallis test for categorical variables to compare the means and proportions of baseline variables between quartiles. The Kruskal–Wallis test was used to assess the association between quartiles of all BNP concentrations. Restricted cubic spline curves were used to visualize OR differences in ROSC, survival to hospital discharge, and neurological outcomes according to BNP. All analyses were performed using SPSS ver. 23 (IBM Corp., New York, NY, USA) and R statistical software (version 3.6.3; R Foundation for Statistical Computing, Vienna, Austria).

## 3. Results

### 3.1. Baseline Characteristics

**Figure 1:**
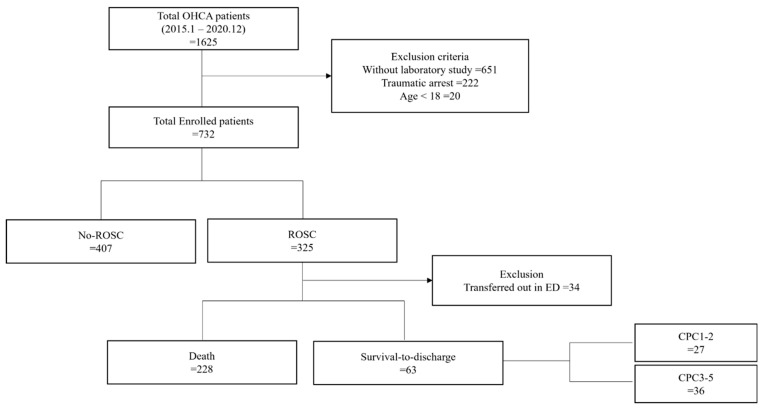
Flowchart of patient screening and selection during the study enrollment process. Abbreviations: OHCA; out-of-hospital cardiac arrest, ROSC; return of spontaneous circulation, ED; emergency department; CPC; cerebral performance category.

During the study period, a total of 1625 patients with OHCA were enrolled. Among them, we excluded cases without BNP (651 cases), cases of traumatic cardiac arrest (222 cases), and cases of patients under 18 years of age (20 cases). Eventually, 732 patients were enrolled in our study. However, patients who were discharged from the ED to a nursing home or home with unknown secondary outcomes were also excluded (Figure 1).

The baseline characteristics are presented in Table 1. Of a total of 732 patients, 325 achieved ROSC. Patients with ROSC were younger than the No-ROSC patients (*p* = 0.002). There was no statistical difference in the presence of hypertension, diabetes mellitus, previous coronary artery disease, cerebral vascular disease, or heart failure between patients in the ROSC group and the No-ROSC group. Among the patients with ROSC, witnessed arrest was higher than in the No-ROSC group (*p* = 0.001), and initial shockable rhythm was also more than in the No-ROSC group (*p* = 0.017). The out-of-hospital CPR time of patients with ROSC was shorter than the No-ROSC group (*p* = 0.000). In-hospital CPR time was also shorter in the ROSC group than in the No-ROSC group (*p* = 0.000). In addition, the total CPR time of patients with ROSC was shorter than the No-ROSC group (*p* = 0.000). The total adrenaline dose (mg) with ROSC was more than the No-ROSC group (*p* = 0.000). BNP was 171.8 (5–5000) pg/mL in the ROSC group, higher than 127.71 (5–5000) in the No-ROSC group, and statistically significant (*p* = 0.007).

Among the patients with ROSC, 63 patients survived to discharge. Patients who survived to discharge had a mean age of 57 years (range: 22–87 years), which was significantly lower than that of those who died during treatment in the hospital (*p* = 0.000). There were no statistical differences in the presence of hypertension, diabetes, coronary artery disease, cerebrovascular disease, or heart failure between patients in the survival to discharge and death groups. In addition, they had a significantly higher rate of initial shockable rhythm (*p* = 0.002). The out-of-hospital CPR time of patients with survival to discharge was shorter than the death group (*p* = 0.005). In-hospital CPR time was also shorter in the survival to discharge group than in the death group (*p* = 0.004). The total CPR time in the group who survived to discharge was shorter than the death group (*p* = 0.000). The total adrenaline dose in the survived-to-discharge group was significantly lower than the death group (*p* = 0.007). The mean BNP concentration was 99.70 pg/mL (range 5–3040.68 pg/mL) in the survival to discharge group, which was significantly lower than 197.88 pg/mL (range 5–5000 pg/mL) observed in the death group (*p* = 0.012).

Twenty-seven patients showed a good neurologic outcome corresponding to a CPC score of 1–2 in the tertiary outcome assessment. There were no statistical differences in the presence of hypertension, diabetes, coronary artery disease, cerebrovascular disease, or heart failure between patients with good neurologic outcomes and those with poor neurologic outcomes. These patients were significantly younger than those with a poor neurologic outcome (*p* = 0.004), were more likely to have an initial shockable rhythm (*p* = 0.001), and the mean total CPR duration was shorter (*p* = 0.021). The out-of-hospital CPR time of patients with good neurologic outcomes was shorter than the poor neurologic group (*p* = 0.001). However, in-hospital CPR time was not statistically different.

### 3.2. Characteristics According to the Quartile of BNP Concentration

Patient characteristics according to the quartile of BNP are presented in Table 2. The BNP concentrations ranged from 5 to 5000 pg/mL, with a mean (±SD) of 419.93 ± 148.09 pg/mL, a median of 148.09 pg/mL, and 25th and 75th percentile values of 48.88 and 426.89 pg/mL, respectively. Patients with high BNP concentrations were older than those with lower concentrations, were likely to be women, and had a history of hypertension, previous coronary disease, previous heart failure, or diabetes mellitus (*p* for trend = 0000). Out-of-hospital CPR time and total CPR time were longer at lower BNP concentrations (*p* for trend = 0.000). In-hospital CPR time was not statistically significant. However, ROSC was more frequent at higher BNP concentrations (*p* for trend = 0.006). Survival to discharge was higher at lower BNP concentrations (*p* for trend = 0.011). SCA of cardiac etiology was more frequent at higher BNP concentrations (*p* for trend = 0.000).

### 3.3. Outcomes after Sudden Cardiac Arrest

**Figure 2:**
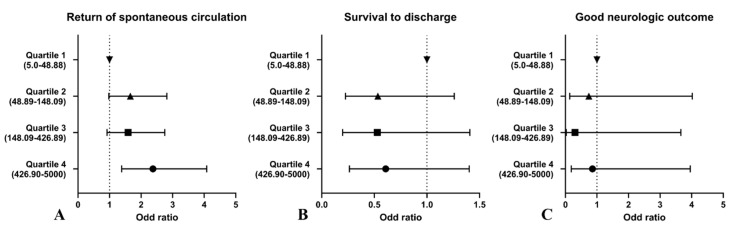
Adjusted odds ratio and 95% confidence intervals of quartile of BNP for outcomes from multivariable models; (**A**) Primary outcome (ROSC); (**B**) Secondary outcome (Survival to discharge); (**C**) Tertiary outcome (Good neurologic outcome).

**Figure 3:**
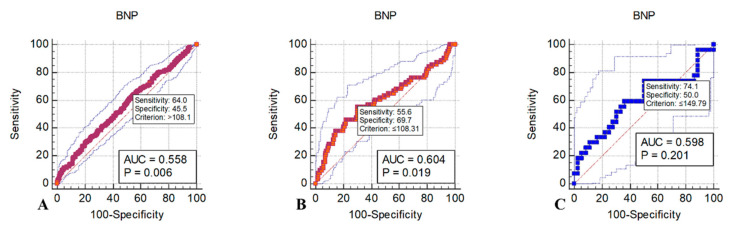
ROC curve and optimal cutoff values of the BNP; Abbreviations: BNP; B-type natriuretic peptide, AUC; area under curve. (**A**) Primary outcome (ROSC); (**B**) Secondary outcome (Survival to discharge); (**C**) Tertiary outcome (Good neurologic outcome).

**Figure 4:**
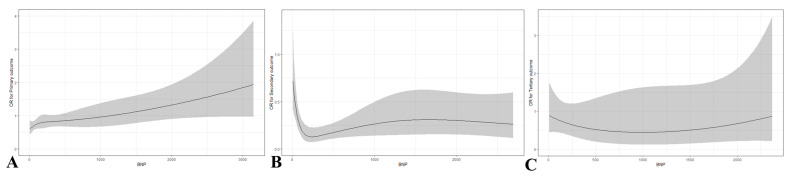
The trend of the odds ratio on outcomes followed by BNP; (**A**) Primary outcome (ROSC); (**B**) Secondary outcome (Survival discharge); (**C**) Tertiary outcome (Good neurologic outcome).

Multivariable logistic regression analyses to the quartile of BNP are presented in Table 3. In the primary outcome, the adjusted OR of BNP was calculated by using age, witnessed arrest, initial shockable rhythm, total CPR duration, and total adrenaline dose. Compared to the group of quartile 1, which is the reference group, the quartile 4 group was identified as an independent factor for ROSC. The adjusted odds ratios for ROSC in the second, third, and fourth quartiles of BNP concentration were 1.660 (95% confidence interval (CI), 0.979–2.815), 1.591 (95% CI, 0.921–2.750), and 2.375 (95% CI, 1.383–4.078), respectively. In the secondary and tertiary outcomes, BNP was not an independent factor. Figure 2 shows the adjusted odds ratio and 95% confidence intervals of BNP for primary, secondary, and tertiary outcomes generated using multivariable models. Figure 3 shows the optimal cutoff values and diagnostic accuracy of the BNP concentration regarding outcomes. Cutoff values of >108.1 pg/mL (AUC: 0.558, 95% CI: 0.522–0.595), ≤108.31 pg/mL (AUC: 0.604, 95% CI: 0.549–0.661), and ≤149.79 pg/mL (AUC: 0.598, 95% CI: 0.467–0.719) for the BNP were predictive of primary, secondary, and tertiary outcome, respectively. A cubic spline curve was fitted to visualize the difference in the odds ratio of clinical outcomes according to BNP level. The odds ratio of the primary outcome increased proportionally to the BNP level. However, the odds ratio of the secondary outcome decreased proportionally to the BNP level. The odds ratio of the tertiary outcome was not correlated with the BNP level. (Figure 4).

## 4. Discussions

In our study, we investigated the correlation of BNP levels measured during ACLS with the clinical outcomes of SCA, irrespective of the etiology of the arrest. We confirmed that ROSC was frequent in patients with high BNP levels measured in the ED, and survival to discharge was frequent in patients with low BNP levels. However, the prognostic value of lower BNP levels regarding good neurologic outcomes was not statistically significant. It is thought that the higher the BNP, the higher the SCA rate of cardiac origin, and for this reason, the higher the ROSC rate, so there may be confounding variables. In addition, BNP was not an independent predictor of outcome, and the AUC was not high. It is not a specific marker needed for outcome assessment.

In previous studies, the value of BNP was primarily investigated in regard to patients with SCA of cardiac origin. In these studies, elevated concentrations of BNP were predictive of an increased risk of death in the hospital and unfavorable neurologic outcomes at the time of hospital discharge [21,22]. In 109 patients, a favorable neurological outcome was predicted if the BNP, measured within one hour of arriving at the emergency room, was lower than 80 pg/mL [21]. Moreover, in a study of 155 patients, high BNP levels measured at hospital admission were associated with 6-month neurologic outcomes and survival to discharge [18]. In contrast, our study included patients with SCA irrespective of the etiology and was relatively large, as 732 patients were enrolled.

BNP is a biomarker whose value as a risk predictor has been documented for various diseases other than cardiac arrest [23]. The efficacy of BNP in predicting SCD in patients with hypertrophic cardiomyopathy has been demonstrated [24]. An association between elevated BNP levels and the development of malignant ventricular arrhythmia, or SCD, has been demonstrated in previous studies [25]. BNP level has been shown to increase as a result of ventricular dilatation, hypertrophy, and fibrosis, resulting in tissue fibrosis and other myocardial arrhythmia-related changes [26]. In another study, BNP was also found to be an independent predictor of SCD in a high-risk group of patients suffering from heart failure with reduced ejection fraction [27]. In one study, it was confirmed that the risk of developing malignant ventricular arrhythmias such as ventricular fibrillation or long-term mortality was lowered when the BNP levels decreased by more than one-third of the baseline value [28].

In a previous prospective study, when 30-day mortality was the measured outcome, NT-pro BNP was high in non-survivors. However, it was confirmed that it was not a significant factor in multivariate analysis [29]. In another prior study, the NT-pro BNP levels of 155 OHCA shockable rhythm patients were measured after 24 h and provided improved risk assessment of poor outcomes after one year, on top of the established risk indices [30]. NT-pro BNP level fluctuates faster than that of BNP and seemingly has superior prognostic value, especially for prognosis in young subjects and female patients [31]. Initial measurements of NT-pro BNP levels may help identify OHCA patients with clinically asymptomatic heart failure or coronary artery disease who require special treatment [25]. Since BNP is rapidly removed from the receptor NPR-C on the surface of target cells, its half-life is very short, about 20 min, whereas NT-pro BNP, which is excreted by the kidneys, has a relatively long half-life of nearly two hours [32]. 

In previous studies, patients with arrests of cardiac origin demonstrated higher ROSC, survival to discharge rates, and better neurologic outcomes when compared with arrests of noncardiac origin [6,33]. In our cohort, it is possible that the inclusion of all SCAs, irrespective of etiology, served as a confounding variable. Nevertheless, since it is challenging to identify the etiology of SCA during ACLS in the actual field, these initial BNP levels can be used for risk stratification. Furthermore, unlike these aforementioned studies, in our multicenter gathered data, the higher BNP concentration correlated with an increased ratio of ROSC. However, the predictive efficacy of BNP has an AUC of 0.558, rendering it an unreliable predictive factor. In addition, in the case of cardiac origin, BNP tends to be higher than that of non-cardiac origin, and since ROSC is achieved more, this may have been a confounding variable. The odds ratio of survival to discharge by BNP measured during ALCS tended to decrease proportionally, which was consistent with the results of the previous study. In determining the staged prognosis of cardiac arrest patients, various scoring systems have been devised for different stages of cardiac arrest. The leading scoring system for predicting ROSC is the RACA score. It is not easily applied in real-world emergency departments due to a large number of variables, including gender, age, etiology, bystanders, location, initial rhythm, and EMS response time [34]. Another score is the Cardiac Arrest Hospital Prognosis (CAHP) score. This is designed for patients admitted to the intensive care unit and is used to predict neurological prognosis. The CAHP score consists of non-shock rhythm, arterial pH, age, arrest settings, no-flow time, low-flow time, and the dose of epinephrine administered at arrest and is used in intensive care units [35]. These various scoring systems do not consist of a single variable, and therefore, predicting the outcome of cardiac arrest patients based on a single test should be avoided [11,36]. In this study, the BNP level alone was not able to predict the outcome of cardiac arrest patients and should be used in conjunction with other various scoring systems.

The study had several limitations. First, the time of BNP measurement was different for all patients. All BNP levels were measured according to cardiopulmonary resuscitation (CPR) guidelines within at least 2–3 cycles during CPR. However, caution is required when interpreting our results because the duration of OHCA and CPR were different. Second, unlike most previous studies, this study did not include follow-up data on BNP levels, and BNP values over time were not provided.; therefore, the No-ROSC group could also be included. Further follow-up studies are needed to properly establish the value of our results. Third, BNP is highly correlated with the patient’s medical history; as such, it may be elevated in cases of chronic kidney disease, previous heart failure, or myocardial infarction. Fourth, as a multicenter study, the BNP measurement method may have been different from that of NT-pro BNP measurement methods in different periods and institutions. Fifth, the relatively small sample size may be biased by the difficulty of obtaining biomarkers performed during ACLS in all out-of-hospital cardiac arrest patients. Sixth, due to the inclusion of a large number of unwitnessed cardiac arrest patients, the timing of resuscitation from the onset of cardiac arrest is unclear. This may affect the results of biomarkers performed during ACLS. Therefore, our results may be biased because all OHCA patients could not be included.

## 5. Conclusions

In patients with SCA of all origins, low BNP concentration measured during ACLS correlated with an increased ratio of survival to discharge. However, BNP measured during ACLS was not found to be an independent factor; therefore, it may not be a suitable predictive factor for risk stratification in SCA of all origins.

## Figures and Tables

**Table 1:** Baseline characteristics of patients.

	Primary Outcome			Secondary Outcome			Tertiary Outcome		
	No-ROSC (*n* = 407)	ROSC (*n* = 325)	*p*-Value	Death (*n* = 228)	Survival to Discharge (*n* = 63)	*p*-Value	Poor (*n* = 36)	Good (*n* = 27)	*p*-Value
Age (years)	75 (19–99)	67 (20–96)	0.002	70 (20–96)	57 (22–87)	0.000	65.5 (22–87)	50 (29–82)	0.004
Male sex, *n* (%)	251 (61.7%)	190 (58.5%)	0.421	131 (57.5%)	42 (66.7%)	0.241	25 (69.4%)	17 (63.0%)	0.787
Hypertension, *n* (%)	192 (47.2%)	138 (42.5%)	0.231	99 (43.0%)	25 (39.7%)	0.745	17 (47.2%)	8 (29.6%)	0.249
Diabetes mellitus, *n* (%)	115 (28.3%)	100 (30.8%)	0.509	67 (29.4%)	20 (31.7%)	0.836	13 (36.1%)	7 (25.9%)	0.558
Previous coronary artery disease, *n* (%)	50 (12.3%)	32 (9.8%)	0.357	24 (10.5%)	6 (9.5%)	1.000	3 (11.1%)	3 (8.3%)	1.000
Previous cerebral vascular disease, *n* (%)	46 (11.3%)	29 (8.9%)	0.351	22 (9.6%)	4 (6.3%)	0.573	1 (3.7%)	3 (8.3%)	0.823
Previous heart failure, *n* (%)	22 (5.4%)	18 (5.5%)	1.000	15 (6.6%)	1 (1.6%)	0.220	0 (0%)	1 (2.8%)	1.000
Bystander CPR, *n* (%)	212 (52.1%)	189 (58.2%)	0.118	127 (55.7%)	39 (61.9%)	0.461	21 (58.3%)	18 (66.7%)	0.680
Witnessed, *n* (%)	192 (47.2%)	196 (60.3%)	0.001	133 (58.3%)	41 (65.1%)	0.411	22 (61.1%)	19 (70.4%)	0.620
Initial shockable rhythm, *n* (%)	27 (6.6%)	39 (12.0%)	0.017	22 (9.6%)	16 (25.4%)	0.002	3 (8.3%)	13 (48.1%)	0.001
Out-of-hospital CPR time (min)	27 (1–174)	21 (1–75)	0.000	22 (1–75)	12 (1–68)	0.005	20 (1–52)	4 (1–68)	0.001
In-hospital CPR time (min)	24 (1–179)	9 (1–102)	0.000	11 (1–102)	7 (1–35)	0.004	7 (2–35)	7 (1–33)	0.686
Total CPR time (min)	52 (3–194)	31 (2–134)	0.000	32.5 (2–134)	25 (2–72)	0.000	29 (3–67)	13 (2–72)	0.021
Total Adrenaline dose (mg)	8 (0–60)	3 (0–34)	0.000	4 (1–34)	3 (0–12)	0.007	3 (1–12)	3 (0–11)	0.622
BNP (pg/mL)	127.71 (5–5000)	171.8 (5–5000)	0.007	197.88 (5–5000)	99.7 (5–3040.68)	0.012	137.45 (5.14–2686.59)	54.75 (5–3040.68)	0.187

Abbreviations: ROSC; return of spontaneous circulation, CPR; cardiopulmonary resuscitation; BNP; B-type natriuretic peptide.

**Table 2:** Characteristics according to the quartile of B-type Natriuretic Peptide Concentration.

	Quartile 1 (*n* = 183) (5.0–48.88 pg/mL)	Quartile 2 (*n* = 183) (48.89–148.09 pg/mL)	Quartile 3 (*n* = 183) (148.09–426.89 pg/mL)	Quartile 4 (*n* = 183) (426.90–5000 pg/mL)	*p*-Value
Age (years)	58 (19–92)	70 (20–95)	78 (24–98)	76 (28–99)	0.000
BNP, mean (pg/mL)	23.62 14.63	93.62 27.40	254.68 79.10	1307.77 1002.45	0.000
Male sex, *n* (%)	133 (72.7%)	108 (59.0%)	102 (55.7%)	98 (53.6%)	0.000
Hypertension, *n* (%)	65 (35.5%)	76 (41.5%)	87 (47.5%)	102 (55.7%)	0.000
Diabetes mellitus, *n* (%)	41 (22.4%)	49 (26.8%)	58 (31.7%)	67 (36.6%)	0.002
Previous coronary disease, *n* (%)	13 (7.1%)	13 (7.1%)	25 (13.7%)	31 (16.9%)	0.004
Previous cerebral vascular disease, *n* (%)	11 (6.0%)	16 (8.7%)	26 (14.2%)	22 (12.0%)	0.051
Previous heart failure, *n* (%)	1 (0.5%)	7 (3.8%)	11 (6.0%)	21 (11.5%)	0.000
Bystander CPR, *n* (%)	116 (63.4%)	95 (51.9%)	89 (48.6%)	101 (55.2%)	0.091
Witnessed, *n* (%)	99 (54.1%)	97 (53.0%)	89 (48.6%)	103 (56.3%)	0.895
Initial shockable rhythm, *n* (%)	24 (13.1%)	18 (9.8%)	6 (3.3%)	18 (9.8%)	0.084
Out-hospital CPR time (min)	27 (1–158)	25 (1–174)	25 (1–93)	22 (1–92)	0.001
In-hospital CPR time (min)	20 (1–179)	17 (1–76)	17 (1–76)	15 (1–93)	0.213
Total CPR time (min)	47 (3–181)	41 (2–194)	41 (3–117)	38 (2–123)	0.000
Total Adrenaline dose (mg)	7 (0–60)	6 (0–26)	5 (1–20)	5 (0–31)	0.235
ROSC, *n* (%)	69 (37.7%)	81 (44.3%)	77 (42.1%)	98 (53.6%)	0.006
Survival to discharge, *n* (%)	24 (13.1%)	13 (7.1%)	9 (4.9%)	17 (9.3%)	0.011
Good neurologic outcome, *n* (%)	11 (6.0%)	7 (3.8%)	8 (4.4%)	10 (5.5%)	0.207
SCA of cardiac etiology, *n* (%)	88 (48.1%)	70 (38.3%)	84 (45.9%)	120 (65.6%)	0.000

Abbreviations: ROSC; return of spontaneous circulation, CPR; cardiopulmonary resuscitation; BNP; B-type natriuretic peptide.

**Table 3:** Multivariable logistic regression analysis to the quartile of B-type Natriuretic Peptide Concentration related ROSC, survival to discharge, and good neurologic outcome.

	Return of Spontaneous Circulation Adjust OR ^a^			Survival to Discharge Adjust OR ^b^			Good Neurologic Outcome Adjust OR ^c^		
Variable	OR	95% CI	*p*-Value	OR	95% CI	*p*-Value	OR	95% CI	*p*-Value
B-type Natriuretic Peptide			0.019			0.442			0.820
Quartile 1 (5.0–48.88 pg/mL)	(reference)			(reference)			(reference)		
Quartile 2 (48.89–148.09 pg/mL)	1.660	0.979–2.815	0.060	0.534	0.226–1.260	0.152	0.743	0.137–4.018	0.730
Quartile 3 (148.09–426.89 pg/mL)	1.591	0.921–2.750	0.096	0.529	0.199–1.409	0.203	0.305	0.025–3.660	0.349
Quartile 4 (426.90–5000 pg/mL)	2.375	1.383–4.078	0.002	0.608	0.264–1.402	0.243	0.857	0.186–3.959	0.844

^a^ Controlling for centered age, witness, initial shockable rhythm, total CPR duration, total adrenaline dose; ^b^ Controlling for centered age, initial shockable rhythm, total CPR duration, total adrenaline dose; ^c^ Controlling for centered age, initial shockable rhythm, total CPR duration; Abbreviations: CI; confidence interval; CPR, cardiopulmonary resuscitation; OR, odds ratio.

## Data Availability

Data cannot be shared publicly because of the involvement of personal information. The data can be accessed with permission from the corresponding author. The contact information is as follows: suffo41@naver.com.
